# Building interprofessional identity in neurology with interactive interprofessional learning: a randomized controlled trial

**DOI:** 10.1186/s12909-025-07492-1

**Published:** 2025-07-01

**Authors:** P. Altmann, B. Fasching, T. Rothschedl, S. Matuschitz, N. Krajnc, J. Ebner, M. Handgraaf, K. Gottfried, P. S. Rommer, T. Berger, M. Wagner-Menghin

**Affiliations:** 1 Department of Neurology, Medical University of Vienna, Waehringer Guertel 18-20, 1090 Vienna, Austria; 2 Comprehensive Center for Clinical Neurosciences and Mental Health, Medical University of Vienna, Vienna, Austria; 3 Vienna General Hospital, Vienna, Austria; 4 Bochum University of Applied Sciences, Bochum, Germany; 5 Rudolf Frey Learning Clinic, University Medical Center of the Johannes Gutenberg University, Mainz, Germany; 6 Department of Psychiatry and Psychotherapy, Medical University of Vienna, Vienna, Austria

**Keywords:** Interprofessional Learning, Neurology Clerkship, Interprofessional Identity, Interprofessional Collaboration, Undergraduate Medical Education

## Abstract

**Background:**

Despite the essential role of interprofessional collaboration in neurology, to improve patient outcomes, targeted research on interprofessional learning (IPL) interventions during neurology clerkships remains limited. This study aimed to assess the impact of a brief interactive IPL workshop on interprofessional identity among medical students.

**Methods:**

In this randomized controlled trial, neurology clerkship students (*N* = 39) were allocated to either a 90-min interactive IPL workshop or a non-interactive control session. We assessed outcomes by triangulating findings across Extended Professional Identity Scale (EPIS-G) scores, challenges and opportunities perceived by students, and reflective responses on interprofessional identity and applicability.

**Results:**

On a group level, The IPL intervention group (*n* = 27) demonstrated improvements in all domains of interprofessional identity on the EPIS-G (paired samples t-test, *p* < 0.001) which was not observed in the control group (*n* = 10). Communication challenges and resource limitations were primary concerns among students, while information sharing and enhanced patient care emerged as key opportunities. Qualitative analysis highlighted students’ increased commitment to collaboration, openness to teamwork, and recognition of the patient care benefits inherent in collaborative practices.

**Conclusions:**

An interactive 90-min IPL workshop within a neurology clerkship can initiate medical students’ interprofessional identity formation. Students' insights into relevant challenges and opportunities indicate their basic understanding of the complexity of collaborative practice. This study supports the future integration of IPL specifically within neurology to advance collaborative practice.

## Introduction

Interprofessional learning (IPL) involves students engaging with perspectives from other health professions [1, 2]. It has gained increasing attention as a key component of healthcare education, particularly in clinical settings that rely on effective teamwork [3, 4]. IPL in neurology fosters understanding, communication, and teamwork, which is critical in neurology and rehabilitation, where patient care is inherently interprofessional [5–7]. As a leading cause of disability and death, neurological disorders highlight the need to develop effective strategies for disease prevention and management. Evidence indicates that interprofessional collaboration (IPC) in neurology may support these efforts [8, 9].

Studies in various healthcare settings have demonstrated that when students of different healthcare professions learn together, collaborative practice is strengthened and patient outcomes improve [10–12]. However, there is limited research on IPL interventions specifically during clinical neurology rotations for medical students, highlighting a need for further exploration [13, 14]. Furthermore, organizing IPL activities face administrative challenges of bringing students of initially different professional curricula together. Also proposed programs require significant time and resources. For example, a 4-h bedside teaching workshop for medical and physical therapy students in a neurology clerkship improved interprofessional communication and clarified professional roles [14]. Similarly, a three-day dementia care workshop for students in nutrition therapy, speech-language pathology, and physiotherapy enhanced self-perceived interprofessional skills, dementia knowledge, and collaborative attitudes [15]. Simulation-based IPL activities in stroke care have also demonstrated the potential to strengthen communication and teamwork across healthcare professions [16]. Although these studies illustrate the benefits of IPL in fostering communication, teamwork, and role clarity, their implementation often required significant resources, such as bedside teaching, simulation, or multi-day workshops. Moreover, few studies have focused on interprofessional learning within neurology clerkships or used controlled designs. In our study, the primary outcome was interprofessional identity, a distinct social identity that encompasses a sense of belonging to, commitment toward, and beliefs about interprofessional collaboration. As defined by extended professional identity theory, it develops alongside profession-specific identity and is considered essential for preparing students to work effectively in interprofessional teams and contribute to collaborative care.

In this mixed-methods study, we explore the potential of an IPL intervention requiring less resources and fewer administrative challenges that might more easily integrate into an existing mono-professional neurology clerkship. We conceived a 90-min interactive workshop for medical students that focused on interprofessional identity development. A control group participated in a non-interactive session for comparison. Quantitative data on interprofessional attitudes were collected retrospectively using a validated pre-post questionnaire. Qualitative insights were derived from reflective questions on interprofessional identity formation and perceived challenges and opportunities of IPC. This approach enabled a detailed examination of how a brief intervention may influence interprofessional identity and short-term changes in professional attitudes within clinical education.

## Methods

### Educational context

The Neurology clerkship at the Medical University of Vienna is a core component of the six-year medical curriculum, taken by students in their fifth year. The five-week clerkship includes one week of neurology lectures, followed by two weeks of interactive teaching seminars and two weeks of clinical practice. For the clinical phase, students are assigned to a ward, where they gain practical experience by engaging with people with neurological diseases under supervision. To the date of this intervention, understanding the challenges and opportunities of IPC and self-reflection in the context of IPL had not been a specified learning objective of this curriculum element. Our IPL intervention was offered as an optional workshop on the last day of the clinical part of their training.

### Study design, participants and randomization

We conducted this randomized controlled trial from March 2024 through June 2024 at the Vienna General Hospital which is our university’s main teaching hospital. There are approximately 330 students enrolled at our university per academic semester. The cohort size in this study reflects the students assigned to the Vienna General Hospital for the clinical part of their clerkship over the course of one semester. There were six groups consisting of 5–8 students each assigned to our site, all of whom were eligible for inclusion in this trial. We offered participation in the interactive workshop in the final week of the clerkship, conducting it on the last day. We collected demographic data (age, gender) from participating students along with their answers to questionnaires and their contribution to the interactive discussion. We performed randomization by preparing six concealed envelopes that would determine the students assignment to a trial group (four envelopes stating “intervention group” and two stating “control group”). Upon the day of the workshop, the workshop coordinator (PA) would randomly draw an envelope that would irrevocably allocate students to their respective group. After this process, the envelope was discarded. Randomization followed a 2:1 ratio. This decision was based on logistical considerations, as six student groups were scheduled during the semester. To maintain feasibility and ensure meaningful data collection from the intervention, we opted for four intervention groups and two control groups. The resulting 2:1 group allocation was implemented at the level of student groups, not individual students. Variation in actual student numbers between groups was due to the natural fluctuation in group size (5–8 students per group), which was not influenced by the allocation procedure. Neither the participants nor the facilitators were blinded to group allocation due to the nature of the intervention. There were no changes to the trial design or protocol once the study began.

### Intervention

The overarching learning outcome of the workshop was to foster an understanding of the relevance of IPC in the clinical practice of neurology. This aim was operationalized through three specific learning outcomes. First, to recognize the importance of interprofessional collaboration, particularly between neurologists and allied health professionals such as physiotherapists and occupational therapists, through the analysis of a clinical case. Second, to reflect on personal experiences with interprofessional teamwork. And, third, to identify and discuss opportunities and challenges of interprofessional collaboration. These outcomes informed our 90-min workshop design, which combined brief lectures alternating with a case discussion, interactive group work, and reflective exercises. The workshop was facilitated by a neurologist (PA), a physical therapist (BF) and an occupational therapist (TR). The session began with brief introductions from the facilitators, followed by a creative icebreaker activity. Students paired up and were encouraged to use colored markers on a piece of paper to illustrate their ideal vision of interprofessional collaboration, responding to the prompt, *"What does ideal interprofessional collaboration look like to you?"*. The resulting drawings showcased imaginative and insightful representations of teamwork, highlighting what the students valued most in interprofessional settings. The main part of the workshop focused on a clinical case involving a 65-year-old man who suffered a stroke and who presented with left-sided hemiparesis, dysarthria and neglect. The neurologist interactively discussed the patient’s acute stroke diagnosis and treatment with the students, while the therapists introduced the International Classification of Functioning, Disability and Health (ICF) framework and detailed the rehabilitation process [17]. After presentations by the occupational therapist and physical therapist, which included a two-minute video showcasing this patient’s rehabilitation process, students engaged in a Think-Pair-Share activity. In this exercise, students worked in pairs to discuss patient cases they had encountered during their clerkship, focusing on interprofessional care and collaboration. They then shared their insights with the larger group, presenting their reflections on how interprofessional teamwork influenced patient outcomes and care delivery. Finally, students engaged in a challenges and opportunities analysis, where they eventually ranked the top three challenges and opportunities related to interprofessional teamwork following a consensus process. At the end of the workshop, students were asked to answer two reflective questions concerning the applicability of knowledge from this workshop and a change in their professional identity.

### Control group

The control group participated in an IPL flipped-class. Subsequent to the facilitators’ introduction, they self-studied a printout of the same educational content used in the intervention: an overview of different professions participating in neurology care, the case presentation, the therapeutic workup (physical therapy and occupational therapy), the ICF framework and rehabilitation process. They had 30 min to read the materials and were subsequently invited to ask questions regarding interprofessional teamwork and the patient rehabilitation process. Facilitators moderated the discussion and answered questions raised by students, but did not contribute questions to the discussion. The control session was intentionally designed as a mono-professional condition without structured activities and was facilitated solely by a neurologist. This setup aimed to minimize contamination (i.e., unintentional exposure to interprofessional content) and create a clear contrast to the interprofessional, interactive intervention. The control group completed the primary outcome assessment in the same format as the intervention group. The control group completed the primary outcome assessment in the same format as the intervention group.

### Outcome measures and data collection

The primary outcome was students'interprofessional identity, assessed using the German version of the EPIS (Extended Professional Identity Scale questionnaire) [18, 19]. The scale is grounded in Extended Professional Identity Theory and assesses three theoretically derived dimensions: interprofessional belonging, commitment, and beliefs. This framework conceptualizes interprofessional identity as a distinct social identity that complements profession-specific identity and supports collaborative behavior. The EPIS consists of 12 items rated on a five-point Likert scale, with higher scores indicating stronger agreement and a more developed interprofessional identity. This results in total scores ranging from 12 to 60, and subscale scores ranging from 4 to 20 for each of the three dimensions. This results in total scores ranging from 12 to 60, and subscale scores from 4 to 20 per dimension. Higher scores reflect a stronger interprofessional identity. While the EPIS-G does not define normative thresholds or cut-off points, higher scores have been empirically linked to increased motivation toward interprofessional collaboration and stronger group performance [20]. This supports its intended use for detecting relative differences over time or between groups [21]. An example question is *“I would be happy to spend the rest of my career with an interprofessional team”*. The German version (EPIS-G) was translated and validated by external collaborators and provided to our institution for the purpose of this study (MH) [19]. The data was collected using a post-then-pre evaluation design. In this approach, students retrospectively rated their pre-workshop perceptions and compared them to their post-workshop perceptions, both of which were assessed simultaneously at the end of the workshop.

Secondary outcomes in the intervention groups included qualitative results from the challenges and opportunities analysis and two reflective questions. As part of the intervention workshop, each student individually listed challenges and opportunities related to interprofessional collaboration, based on their clinical experiences and workshop discussions. They then voted on their top three challenges and opportunities using flashcards numbered 1, 2, and 3, with"3"indicating the highest level of agreement. The three top-ranked challenges and opportunities were documented for each of the intervention groups. For the reflective questions, students were asked*,"Which insights from today’s workshop do you think you can apply to your practice tomorrow?"* and *"How has your own professional identity changed as a result of attending this workshop?"*. We instructed students to provide concise, one-sentence responses, similar to a one-minute summary. For the qualitative analysis, we employed a inductive content analysis [22]. We opted for an inductive approach, allowing themes to emerge from the data without reliance on a pre-existing framework. Three researchers (PA, BF, and JE) independently reviewed the student responses, generating initial codes based on the content of the statements. These codes were then compared and iteratively refined through discussion, leading to a consensus on the final themes.

### Statistical analysis

Statistical analysis was performed using SPSS 29.0 (SPSS Inc, Chicago, IL, USA). Categorical variables were expressed as absolute frequencies and percentages, continuous parametric variables as mean and standard deviation (SD). The primary outcome measure (i.e., EPIS-G score and EPIS-G subscale scores) was analyzed using Wilcoxon signed-rank test. Significance was set at *p* < 0.05, thus Bonferroni adjusted significance level for the EPIS-subscales was 0.05/3 = 0.017.

### Reflexivity

Our author team represents a range of professional backgrounds, including neurology (PA, NK, JE, PSR, TB), physiotherapy (BF, MH), occupational therapy (TR, SM), and medical education (PA, MH, KG, MWM). The workshop was developed by PA, BF, and TR, with didactic input from MWM, and facilitated by PA, BF, and TR. PA, MH, and MWM were responsible for the study design and research methodology. All statistical analyses were conducted by NK, who had no involvement in the workshop. Qualitative data were analyzed by PA, BF, and JE using an inductive approach. We recognize that our positionality as educators, particularly PA’s dual role as both facilitator and primary analyst, may have influenced the interpretation of findings. To mitigate this, we incorporated investigator triangulation, involved professionals from physiotherapy and occupational therapy in the workshop’s conception, delivery, and analysis, and ensured methodological oversight by MWM, who was not involved in content delivery. Furthermore, our mixed-methods design and methodological triangulation across quantitative and qualitative data were intended to enhance the validity of findings and reduce the risk of bias. All results were reviewed and discussed by the full author group to integrate diverse professional perspectives and strengthen the trustworthiness of the analysis.

## Results

### Student characteristics

**Fig. 1:**
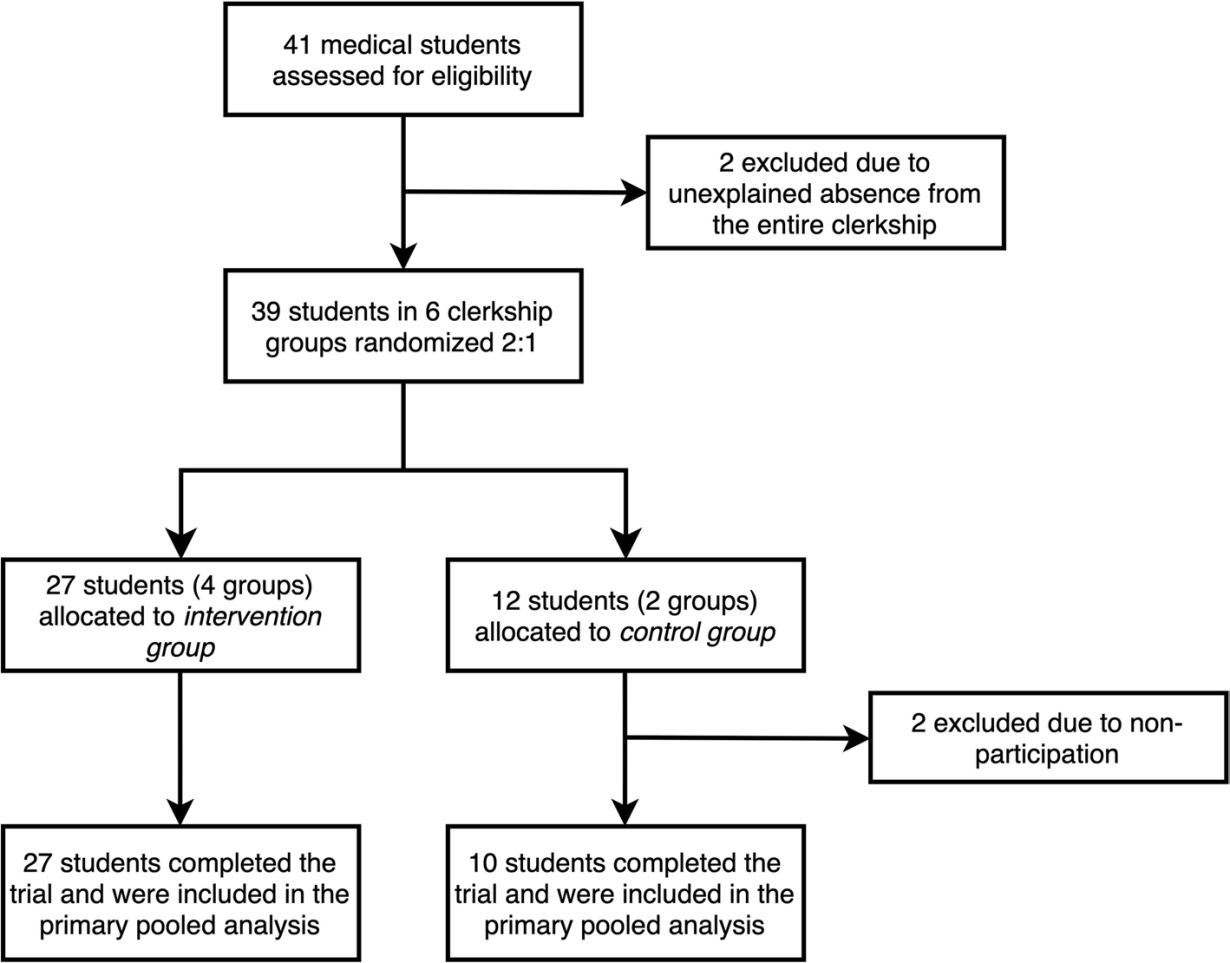
CONSORT flow diagram showing student participation across each phase of this randomized open-controlled trial of interprofessional learning: enrollment, intervention allocation, and final analysis

The data presented in this analysis is pooled from four 90-min interactive intervention workshops and two control sessions. Figure 1 demonstrates the participant flow. A total of 41 students in six groups were assigned to complete their neurology clerkship at our teaching hospital during the study period. Once the workshop began, no participant opted out of the study and all remaining students (27 in the intervention group and 10 in the control group) completed the trial and were included in the final analysis. The mean (SD) age of all participants was 25 (± 2) years. The majority of participants were female, comprising 70.3% of the total cohort. The intervention group had a mean (SD) age of 25 years (± 1), while the control group had a slightly higher mean (SD) age of 27 years (± 3) (*p* = 0.067). Both groups had a similar gender distribution, with 70.4% of the intervention group and 70.0% of the control group identifying as female (*p* > 0.999).

### Interprofessional identity

**Fig. 2:**
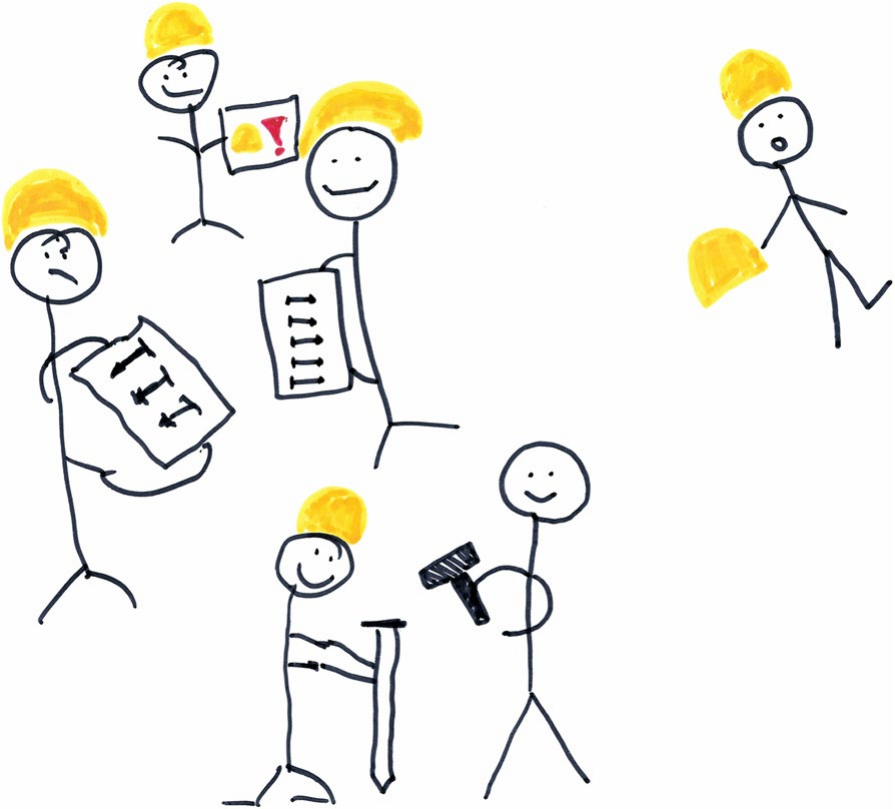
Example student illustration depicting interprofessional collaboration as a construction site, emphasizing teamwork, shared goals, and mutual support

As a first task and ice-breaking exercises, we asked our students to draw an image of what ideal interprofessional collaboration looked like to them. One example from this exercise is illustrated in Fig. 2. The students explained their drawing as an allegory of IPC in healthcare, using a construction site to illustrate essential teamwork dynamics. In their image, healthcare workers from various professions each contribute uniquely: one brings a clear plan to support a colleague facing a challenging task, emphasizing the value of shared knowledge in problem-solving. Another figure focuses on safety, symbolizing holistic patient care, while a team member who forgot their “helmet” receives it from a colleague, showing mutual support in preparing for patient care. Together, these elements, as the students described, illustrate collaborative learning, joint decision-making, and the belief that complex goals are more achievable through teamwork and collective expertise.

**Fig. 3:**
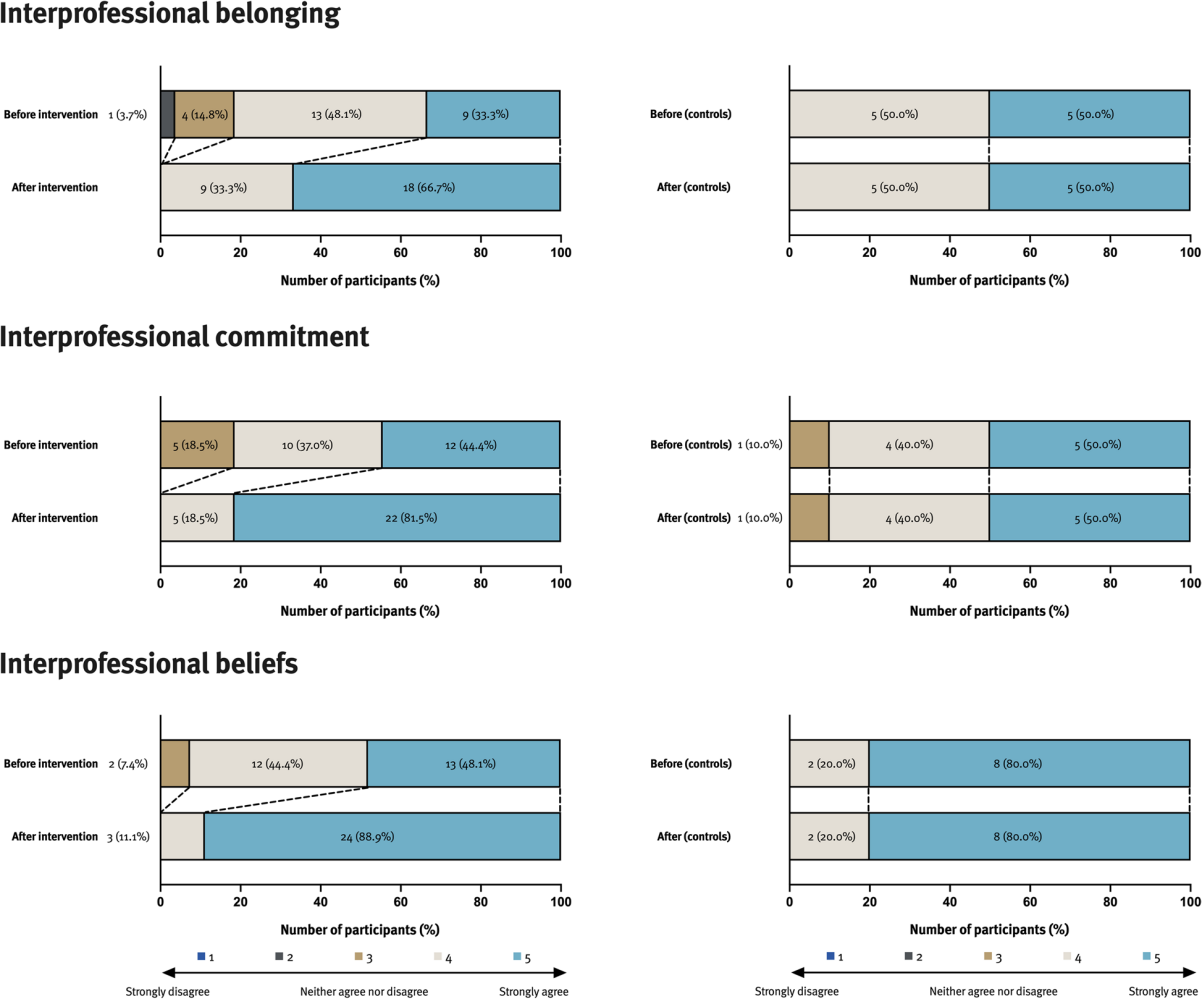
These bar charts plot the mean score per EPIS-G subscale from participating students before and after the IPL workshop (*n* = 27 in the intervention group and *n* = 10 in the control group). Colors indicate the level of agreement on a 5-point Likert scale. EPIS-G: Extended Professional Identity Scale-German, IPL: interprofessional learning

As for our quantitative results, students participating in our intervention perceived improvements on the EPIS-G scale for all three domains of interprofessional identity on a group level (Fig. 3 and Table 1). Overall median (range) EPIS-G scores were significantly higher in the intervention group (58 [47–60]) compared to the pre scores (49 [38–60]) with a large within-group effect size (*r* = 0.81). No significant difference was observed in the control group’s score (54.5 [41–60] vs. 54.5 [41–60], *p* = 0.317).

**Table 1:** Analysis of EPIS-G scores

*Sum Scores per EPIS domain*	Intervention group (*n* = 27)				Control group (*n* = 10)			
	Before^a^	After^a^	*r*	*p-value*^b^	Before^a^	After^a^	*r*	*p-value*^b^
Interprofessional belonging	16 (8–20)	19 (14–20)	0.81	< 0.001	17.5 (15–20)	17.5 (15–20)	0.00	> 0.999
Interprofessional commitment	16 (12–20)	20 (15–20)	0.78	< 0.001	17.5 (12–20)	17.5 (12–20)	0.00	> 0.999
Interprofessional beliefs	17 (12–20)	20 (15–20)	0.76	< 0.001	20 (14–20)	20 (14–20)	0.32	0.317
*Median Scores per EPIS domain*								
Interprofessional belonging	4 (2–5)	5 (4–5)	0.71	< 0.001	4.5 (4–5)	4.5 (4–5)	0.00	> 0.999
Interprofessional commitment	4 (3–5)	5 (4–5)	0.62	0.001	4.5 (3–5)	4.5 (3–5)	0.00	> 0.999
Interprofessional beliefs	4 (3–5)	5 (4–5)	0.65	< 0.001	5 (4–5)	5 (4–5)	0.00	> 0.999
Total EPIS score	49 (38–60)	58 (47–60)	0.81	< 0.001	54.5 (41–60)	54.5 (41–60)	0.32	0.317

*r* Effect size for Wilcoxon signed-rank test (repeated measures), *EPIS-G* Extended Professional Identity Scale (German)

^a^Median (range)

^b^Wilcoxon signed-rank test

### Perceived challenges and opportunities

Students in the four intervention groups individually listed and ranked key challenges and opportunities related to interprofessional teamwork, followed by a consensus process to identify group-level priorities. Across all groups, communication was consistently ranked as the most significant challenge, holding the top position in each group. In three groups, high demands on time and personnel were ranked second. Additional second- and third-ranked challenges included maintaining a consistent flow of information, differentiating professional responsibilities, establishing and maintaining team spirit, planning workflows, and the potential for conflict within interprofessional teams. These challenges highlight concerns both at the level of interpersonal communication and systemic resource constraints.

In terms of opportunities, gaining more accurate and comprehensive information about patients was ranked as the top opportunity in two groups and appeared among the top three in all groups. Improved patient care and outcomes were also consistently ranked in the top three opportunities. Other opportunities included appreciation for other health professions, reducing the workload for individual professions, mutual education and knowledge sharing, increased patient safety, and exposure to different professional perspectives. The concept of interprofessional mutual support was specifically emphasized in several groups. While specific rankings varied, the most consistently valued opportunities focused on enhanced information exchange, improved outcomes for patients, and mutual professional support through collaboration.

### Individual reflections on applicability and professional identity change

At the conclusion of the workshop, students were asked two questions: *“Which insights from today’s workshop do you think you can apply to your practice tomorrow?"* and *“How has your own professional identity changed as a result of attending this workshop?”* Their responses were grouped into key categories which are summarized in Table 2.

**Table 2:** Content analysis of student perspectives on applicability and interprofessional identity

Question 1: Which insights from today’s workshop do you think you can apply to your practice tomorrow?			
Theme	Frequency (n)	Example Quotes	Coding Examples
Improved communication with other professions	10	"Communication is essential for maintaining the flow of information between teams."	Communication, information flow, transparency, listening
Openness to interprofessional collaboration	7	"The exchange of knowledge between professions improves outcomes."	Collaboration, knowledge exchange, teamwork
Benefits of teamwork for patient care	6	"Collaboration leads to better care as each profession brings a unique perspective."	Patient care, professional perspective, patient outcomes
Involving patients in care decisions	3	"Patients should be directly involved in planning their care."	Care planning, patient involvement, patient autonomy
Specific knowledge about neurological care	1	"The Barthel Index is highly relevant for patient management."	Neurology-specific knowledge
Question 2: How has your own professional identity changed as a result of attending this workshop?			
Theme	Frequency (n)	Example Quotes	Coding Examples
Identifying with a collaborative role in team-based care	16	"I now see myself as a crucial part of a team."	Team, identification, collaboration, inclusion
Acknowledging the value and challenges of collaboration	6	"I now have a better grasp of the benefits and challenges of collaboration."	Challenges, benefits
Increasing confidence in collaborative roles	4	"I feel more confident in my ability to collaborate across professions."	Confidence, help, empowerment
No change in professional identity	1	"I was already convinced that good care can't be provided by physicians alone."	No change

Themes, frequencies, and example quotes illustrating practical applications and professional identity shifts reported by participants following the interprofessional learning workshop

For the first question, many students identified improved communication with other professions as a key takeaway. One student noted, *"Clear communication between professions is crucial for achieving the best patient outcomes.".* Others highlighted openness to interprofessional collaboration, recognizing the benefits of working closely with colleagues from different disciplines. Some students also mentioned the benefits of teamwork for patient care, acknowledging that collaboration provided diverse perspectives that led to better patient care. A few emphasized the importance of involving patients in care decisions, with one student saying, *"Involving patients directly in planning their care makes the process more effective."*. Specific knowledge about neurological care, such as the Barthel Index, was also noted by one student as immediately relevant for practice.

Regarding changes in professional identity, most students reported identifying more strongly with a collaborative role in team-based care. They described a shift toward seeing themselves as integral parts of interprofessional teams. One student reflected, *"I now see myself as part of an interprofessional team to which every profession contributes.".* A smaller group acknowledged both the value and the challenges of collaboration, recognizing the complexities of working across disciplines. Some students reported increasing confidence in their ability to collaborate effectively. As one participant stated, *"My confidence in collaborating with other professions has grown."* One student indicated no change in their professional identity, as they had already embraced the importance of teamwork.

## Discussion

Our randomized controlled trial demonstrated the impact of a structured IPL workshop with guided interactive activities on medical students’ interprofessional identity and competencies during their clinical neurology clerkship. By triangulating insights from changes in the EPIS-G scores, the challenges and opportunities task and the applicability and change task, this study provides a nuanced view of how a 90-min educational intervention can spark medical students’ formation of interprofessional attitudes. The EPIS-G scores indicated significant change in all domains for interprofessional identity for the intervention group. Following the three guided interactive activities students rated their initial levels of interprofessional commitment, belonging and beliefs significantly lower than after the intervention. Students of the control group, who were not guided to reflect on the studied material, rated their pre-class level of attitude similar to their post-class level. We attribute this change in attitude to the guided interactive activities the intervention group participated in.

Beyond the quantitative measures, students of the intervention group identified communication and resource management as primary challenges concerning IPC, while highlighting enhanced information exchange and improved patient outcomes as significant opportunities. Furthermore, the applicability and change task provided additional insights into the students’ professional identity development. Many students emphasized the importance of improved communication and the benefits of IPC for patient care. These findings suggest that our IPL intervention was successful in fostering a collaborative mindset, with students increasingly recognizing their role within interprofessional teams and valuing the contributions of diverse professions. Students participating in this trial self-reported relatively high baseline scores on the professional identity scale in both study groups, with even higher scores observed in the control group. This may have influenced the magnitude of the observed differences and warrants further discussion. Other studies reported high baseline scores for scales estimating interprofessional attitudes as well [23, 24]. This observation might be partially attributable to an overestimation of knowledge or capabilities that may have introduced cognitive bias [25, 26]. Importantly, this dynamic may reflect a form of response shift bias, whereby students in the intervention group, prompted by the workshop experience, reassessed and recontextualized their prior attitudes more critically. Their significantly lower retrospective pre-workshop scores suggest that the intervention primarily affected how they understood their baseline interprofessional identity. In contrast, the control group, lacking exposure to these experiences, may not have recognized what they had yet to learn, and thus could not recalibrate their self-assessment in the same way. This interpretation is supported by the observation that post-workshop scores between the two groups were similar. The effect of the intervention may therefore lie less in raising post-workshop identity scores, and more in shifting students’ understanding of their initial attitudes and assumptions. In contrast, students who participated in our workshop likely developed a more granular view of their interprofessional skills, having firsthand experience of what they could learn and where their deficits lay, resulting in relatively lower baseline scores leaving more room for improvement. Addressing these influences in future studies could involve more sensitive, nuanced assessments of attitude change and may require a different method for survey delivery. Another explanation for the high baseline scores in both groups could be the influence of a hidden curriculum [27]. This concept refers to the implicit messages, values, and behaviors that are communicated to learners outside of formal curricula. Implicitly taught aspects of IPC may have been subtly conveyed during earlier stages of training at our institution, thereby priming students with positive attitudes toward teamwork before they engaged with our IPL intervention. Identifying and understanding the impact of a hidden curriculum will be crucial in future studies, as it may play a significant role in shaping the attitudes of healthcare students before they encounter formalized IPL content. Nevertheless, the increase in EPIS-G scores, as demonstrated in response to our intervention workshop, was statistically significant only for the intervention group, not the control group.

In an effort to triangulate our insights from this intervention around quantitative and qualitative results, we believe our study highlights the differential impact of IPL within this 90-min workshop. From a quantitative perspective, the intervention showed an increase in EPIS scores in our intervention group only. The qualitative data from our study provides a valuable angle to understand the nuances behind the quantitative findings. The reflective questions revealed a strong emphasis on improved communication, openness to IPC, and the perceived benefits of teamwork for patient care. These themes resonate well with the significant gains on the EPIS-G, illustrating how our intervention was successful in promoting a collaborative mindset among students. Also, our challenges & opportunities analysis demonstrated our students’ ability to critically reflect on IPC.

Our study’s outcomes align closely with the Framework for Interprofessional Competency Assessment (FINCA), which underscores the importance of observable collaborative activities, interprofessional problem-solving, and fostering shared professional identities [28]. According to the FINCA model, effective interprofessional education should, among others, enhance competencies in communication, teamwork, and collaborative decision-making, which were core components in our educational intervention. The improvement in our students'sense of interprofessional identity aligns with FINCA’s emphasis on fostering a shared team identity among participants from diverse healthcare backgrounds. Specifically, FINCA highlights the importance of information sharing, maintaining communication, and negotiating as central to successful interprofessional practice. This was reflected in our workshop's focus, where students engaged in practical exercises that required active communication and problem-solving whilst being surrounded by an interprofessional team, echoing FINCA’s “observable collaborative activities.”

To our knowledge this is the first study evaluating an IPL workshop as part of a neurology clerkship specifically catering to medical students in a real-life education setting. Strengths of our study include the randomized controlled design and mixed-methods approach, which allowed for a comprehensive analysis of both quantitative and qualitative outcomes. Quantitative data was measured using a validated questionnaire and the triangulation of data provides a more holistic understanding of how structured IPL interventions impact medical students. Scores across all three domains of interprofessional identity correlated well, which suggests that the EPIS-G was an appropriate tool to measure the impact of our workshop [18]. Furthermore, our study was specifically tailored for neurology – a specialty where interprofessional teamwork is a fundamental aspect of clinical practice. Therefore, we believe our conclusions highlight the value of IPL in a medical curriculum in general, not only neurology. Additionally, the alignment of our findings with the ideas behind FINCA supports the reliability of our results, indicating that our data is consistent with an established interprofessional framework. Our 90-min IPL intervention is notable for its brevity and ease of integration into existing curricula, distinguishing it from previous studies that often involve longer or more resource-intensive formats, Nevertheless, several limitations should be acknowledged. High pre-intervention scores on the EPIS-G self-report may have masked more subtle changes in students who started with high baseline scores, leading to skewed estimates of our intervention particularly in comparison to our control group. Additionally, the post-then-pre design, while advantageous in controlling for memory bias, could have introduced retrospective bias as students evaluate their attitudes in hindsight. Another limitation of this study is that the intervention comprised multiple components delivered in combination, making it difficult to determine which specific elements were most impactful. Future studies may consider dismantling or comparing components to identify those most strongly associated with interprofessional identity development. Furthermore, secondary outcomes were only collected in the intervention group. This decision stemmed from our intention to keep the control group passive in order to preserve contrast and avoid contamination. Nonetheless, collecting these outcomes in both groups would have enabled more direct comparisons, may have provided greater depth, and should be considered in future studies. Future studies should also consider alternative evaluation methods, such as separate pre and post measurements, longitudinal follow-ups or observational assessments, to address these limitations. Moreover, students participating in this trial represented a “convenience sample” as all students assigned to our institution from one semester were eligible to participate, thus, limiting our results in terms of sample size and generalizability. Also, 70% of our participants identified as females, which could have introduced gender bias.

While our intervention focused on medical students, it was designed and delivered by an interprofessional team and could be expanded into a full interprofessional education format. Including learners from other health professions, such as physiotherapy and occupational therapy, may enrich the experience and promote shared identity formation. Our findings suggest that medical students are ready for such engagement, having demonstrated an interprofessional mindset, reflected on collaboration, and expressed a sense of belonging in team-based care. Future studies could build on these insights by directly comparing single-profession and interprofessional learning environments, examining not only outcomes but also how students construct meaning and professional identity within interprofessional teams. Scaling this model across institutions or specialties may offer further insight into how brief, structured IPL interventions support collaborative readiness in clinical training. Future research should also examine how changes in interprofessional identity influence observable behaviors in clinical practice, helping to link attitudinal development with collaborative performance. Combining identity measures with observed teamwork performance may help clarify how identity shapes real-world collaboration.

In conclusion, this study shows that a brief, structured IPL workshop embedded in a neurology clerkship can enhance medical students’ interprofessional identity and understanding of collaborative practice. Students demonstrated an ability to identify challenges, such as communication and resource management, while appreciating the benefits of teamwork for patient care. These findings address gaps in IPL research by demonstrating that short, targeted interventions can yield measurable educational outcomes without requiring extensive resources. It seems our workshop equipped students with both the motivation and practical skills necessary for teamwork in complex clinical environments. Future studies should explore the scalability of such interventions, their long-term effects on interprofessional competencies, and their impact on patient care in diverse healthcare settings. They should prioritize skills like communication and resource management to ensure that healthcare professionals are not only motivated but also well-equipped for complex teamwork in clinical settings.

## Data Availability

The datasets used and/or analysed during the current study are available from the corresponding author on reasonable request.
